# A novel understanding of the nature of epistemic vice

**DOI:** 10.1007/s11229-022-03519-y

**Published:** 2022-02-28

**Authors:** Alkis Kotsonis

**Affiliations:** School of Education, College of Social Sciences, University of Glasgow, St. Andrews Building, G3 6NH Glasgow, United Kingdom

**Keywords:** Intellectual vice, Obstructivism, Motivationalism, Epistemic harm, Non-obstructing, excess-motivation vice

## Abstract

My aim in this paper is to present and discuss a novel understanding of the nature of epistemic vice. I highlight that epistemic vice such as excessive curiosity, gossip and excessive inquisitiveness do not obstruct the acquisition, transmission and retention of knowledge and are not characterized by a deficiency of epistemic desires or vicious epistemic motivations. However, I argue that such traits ought to be classified as epistemic vices because the agent who possesses them causes epistemic harm to other agents through those traits’ characteristic activities.

To remedy obstructivism’s inability to account for vices that cause epistemic harm in other ways besides blocking effective epistemic inquiry, I propose an amended version of this theory. I argue that epistemic vices are character traits, attitudes, and ways of thinking that obstruct the acquisition, transmission, and retention of knowledge and/or cause other kinds of epistemic harm. In addition, I propose a modified version of motivationalism that cashes out non-obstructing, excess-motivation vices in terms of motivation simply by acknowledging, and incorporating into theory, excessive epistemic drives and the negative epistemic (and non-epistemic) consequences stemming from them.

## Introductory remarks

Vice epistemology is the study of the “nature, identity and epistemological significance of intellectual vices” (Cassam, 2016, p. 159). It involves identifying and analyzing certain character traits, attitudes and/or modes of thinking that can account for imperfect epistemic states (e.g., false beliefs, deficiency of epistemic goods, etc.). One influential approach to the study of epistemic vice^1^ is motivationalism which is informed by the responsibilist understanding of the concept of intellectual virtue (see e.g., Code 1984; Montmarquet, 1993; Zagzebski, 1996; Baehr, 2009, 2011). For virtue responsibilists, intellectual virtues (such as open-mindedness and intellectual courage) are characterized by the agent’s strong desire for the acquisition of epistemic goods (e.g., truth and knowledge). Scholars working in motivationalism uphold the inversion thesis according to which vices are the exact opposite of virtues—meaning that they are characterized by imperfect epistemic motivations (see Crerar 2018, p. 754). For them, intellectual vices stem from a lack of epistemic drives (i.e., one is not interested in acquiring epistemic goods) or even an explicit antagonization of epistemic goods (i.e., one willingly promotes epistemic falsehoods). Scholars such as Zagzebski (1996), Montmarquet (2000) and Baehr (2010) understand intellectual vices as involving motivational defects—i.e., motivations which lack epistemic value either because they are deficient or because they are outright vicious. For example, they maintain that a closed-minded agent is characterized by her lack of epistemic desires or, in some cases, her explicit dislike of epistemic goods.

Scholars such as Cassam (2016, 2019) and Crerar (2018) have recently discussed, contra-motivationalism, examples of epistemic vices which are not characterized by imperfect epistemic motivations. Such examples show that an agent can possess epistemic vices despite having a strong motivation for obtaining epistemic goods. This undermines the motivationalist view on vice and has led Cassam (2019) to propose a novel view on epistemic vice called obstructivism. Cassam puts forward a consequentialist view on vice and maintains that vices are traits, attitudes, and way of thinking that systematically obstruct the acquisition, transmission, and retention of knowledge (Cassam, 2019, p. 1). For example, the character trait of closedmindedness is an epistemic vice because of its negative epistemic consequences—it hinders effective epistemic inquiry.

My aim in this paper is to argue that neither the theory of motivationalism nor the theory of obstructivism are broad enough to account for non-obstructing, excess-motivation epistemic vices such as excessive curiosity, gossip, and excessive inquisitiveness. These are traits which do not obstruct the acquisition, transmission, and retention of knowledge (hence non-obstructing) nor involve deficient or vicious epistemic motivations (they are characterized by excess epistemic desires). Nonetheless, I argue that such traits should be classified as epistemic vices since, through their characteristic activities, the agents who possess them come to inflict epistemic harm to other people. To account for such traits, I present and discuss a novel understanding of the nature of epistemic vice: I develop and propose a broadened version of obstructivism which maintains the original theory’s consequentialist nature and incorporates epistemic vices which cause non-obstructing epistemic harm. In addition, I propose a modified version of motivationalism that can cash out non-obstructing, excess-motivation intellectual vices in terms of motivation simply by acknowledging, and incorporating into theory, excessive epistemic drives and the negative epistemic (and non-epistemic) consequences stemming from them.

In what follows (Sect. 2), I begin with a discussion of Cassam’s (2016, 2019) and Crerar’s (2018) arguments against motivationalism and briefly outline Cassam’s (2019) obstructivism theory. Then, in the third section of this paper, I proceed to discuss excessive curiosity, gossip and excessive inquisitiveness and show that neither obstructivism nor motivationalism can accommodate (in their current form) such non-obstructing, excess-motivation epistemic vices. To remedy this, I then move on in the fourth section of this paper, to develop and propose broader versions of both the theory of obstructivism and the theory of motivationalism that retain the essential characteristics of the original theories while incorporating non-obstructing, excess-motivation epistemic vices under their conceptualization of the term of epistemic vice.

## The theory of obstructivism

Scholars who follow the motivationalism viewpoint maintain that intellectual vices “involve non-instrumental motives to oppose, antagonize, or actively avoid things that are epistemically good in themselves” (Tanesini, 2018, p. 350). For instance, Battaly (2017, p. 5) argues that intellectual vices are “partly composed of bad epistemic motives”. Still, not everyone agrees with the motivationalist view on vice. For instance, Crerar (2018) has recently discussed the case of Oblomov and provided evidence against what he calls the presence conception of intellectual vice (i.e., the view according to which intellectual vices require the presence of vicious motivations). The Oblomov case shows that one can possess epistemic vice without having vicious epistemic motivations. According to Crerar (2018), Oblomov possesses the epistemic vices of laziness and incuriosity even though he lacks vicious epistemic motivations—he is simply uninterested in obtaining epistemic goods. Examples such as this show that an explicit antagonization and opposition towards epistemic goods is not a necessary condition for epistemic vice.

Still, most scholars following motivationalism believe that epistemic vices are characterized by a lack of epistemic drives and do not require an explicit antagonization or opposition of epistemic goods (see e.g., Montmarquet 2000; Baehr, 2010). According to this view, an agent does not need to have bad epistemic motivations to possess epistemic vice—epistemic vice simply presupposes “a lack of desire for knowledge” (Baehr, 2010, p. 209). This view offers a good enough account of epistemic vices (such as laziness and incuriosity) that are not characterized by vicious motivations.

This view does a competent job at explaining in motivational terms vices such as laziness and incuriosity—i.e., epistemic vices which are not characterized by vicious motivations. Crerar (2018) calls this motivationalist view (i.e., the view according to which intellectual vices presuppose the absence of good motivations) the absence conception of epistemic vice and discusses the case of Galileo to object to it. Galileo is often depicted in the literature of vice epistemology as a closed-minded individual (see e.g., Roberts and Wood, 2007, p. 254) and this closemindedness is partly attributed to Galileo’s feelings of intellectual superiority. Galileo’s closemindedness does not involve the presence of vicious epistemic motivations nor the lack of epistemic drives—Galileo was highly motivated in his epistemic pursuits due to his strong desire to acquire epistemic goods. Crerar’s (2018) discussion of Galileo illustrates that not all epistemic vices are characterized by the absence of good epistemic motivations (or presence of vicious ones).

Still, Crerar (2018) was not the first to come up with such examples. Before him, Cassam (2016) had already discussed the case of a conspiracy theorist called Oliver who—despite having strong motivations to acquire the truth—is characterized by his gullibility. Examples such as the ones discussed in this section led Cassam to develop and propose a novel understanding of epistemic vice, one that is similar to Driver’s (2001) consequentialist view on moral virtue and vice (Cassam, 2019, p. 11). Cassam argues that epistemic vices are ‘blameworthy, or otherwise reprehensible character traits, attitudes or ways of thinking that systematically obstruct the gaining, keeping or sharing of knowledge’ (Cassam, 2019, p. 1). For Cassam, the presence of epistemic vice is not conditioned on the agent having imperfect epistemic motivations: “epistemic vices aren’t rooted in a desire for ignorance and needn’t have epistemic motives that account for their badness” (Cassam, 2016, p. 166). Cassam’s understanding of vice is a consequentialist one. He characterizes epistemic vices in term of their negative consequences for effective epistemic inquiry (Cassam, 2019, p. 5).

As is evident from his definition of intellectual vice, Cassam does not limit his discussion to character traits (e.g., gullibility, closedmindedness) but expands his understanding of the concept of epistemic vice to include various attitudes (e.g., epistemic prejudice and epistemic malevolence) and ways of thinking (e.g., biased thinking, superstitious thinking). For Cassam, thinking vices are distinct from character vices in that the former are not necessarily part of the latter. For instance, an agent may succumb to closed-minded thinking under certain conditions (e.g., when talking to her mother) but that does not necessarily mean that they possess the vice of closed-mindedness. It may very well be that in the majority of cases (e.g., when she is not talking to her mother) the agent is open-minded. Cassam (2019, p. 56) argues that in order for an agent to possess the trait of closed-mindedness, one needs to consistently engage in closed-minded thinking.

Cassam also discusses epistemic attitudes as a kind of intellectual vice and notes that attitudes “are orientations or postures towards something” (2019, p. 13). For him, epistemic attitudes and character traits are closely related but distinct. To distinguish between attitudes and traits, Cassam (2019, p. 98) notes that “it makes perfect sense to suppose that a person might display a particular attitude in response to a particular question even if they lack the corresponding character trait; their attitude in this case might be out of character”. For instance, the fact that one might exhibit an intellectually arrogant attitude in a given scenario or epistemic domain does not necessarily entail that they possess the character trait of arrogance. It could be that they have an intellectually arrogant attitude only under certain circumstances. One of Cassam’s (2019, p. 94) own examples is the arrogant attitude of certain senior members of the Bush administration that “prevented them from coming to know how many troops would be needed in Iraq”.

## Non-obstructing, excess-motivation epistemic vices

### Excessive curiosity, gossip and excessive inquisitiveness

In this section, I proceed to present examples of epistemic vices which do not obstruct effective epistemic inquiry (hence are non-obstructing) nor involve deficient or vicious epistemic motivations. I discuss the cases of (i) excessive curiosity, (ii) gossiping and (iii) excessive inquisitiveness and argue that traits such as these should be classified as epistemic vices due to their negative epistemic consequences^2^. Through the characteristic activities of these epistemic vices, agents cause epistemic harm to other people^3^.

Epistemic harm has a central role in my understanding of the concept of epistemic vice. The kind of epistemic vices discussed in this section are characterized by the epistemic harm that the agent inflicts to other agents through those vices’ characteristic activities. Following Cassam’s (2019) conception of vice, I argue that such traits are intellectual vices because of the negative epistemic consequences they bring about. My discussion is informed by Fricker’s (2007) understanding of epistemic injustice. Fricker develops the concept of epistemic injustice to classify cases of injustice that are distinctively of an epistemic kind. For her, epistemic injustice “consisting, most fundamentally, in a wrong done to someone specifically in their capacity as a knower” (Fricker, 2007, p. 1)^4^. In her 2007 book titled ‘*Epistemic Injustice: Power and the Ethics of Knowledge*’, Fricker identifies two kinds of epistemic injustice: (a) testimonial injustice and (b) hermeneutical injustice. Briefly put, testimonial injustice occurs when a hearer does not give the appropriate credence to a speaker’s word (and hence the speaker is wronged in their capacity as a knower—Fricker 2007, p. 10) and hermeneutical injustice occurs when one cannot make sense of their social experiences due to “prejudicial flaws in shared resources for social interpretation” and hence prevents the knower from understanding and communicating their experiences (Fricker, 2007, p. 148)^5^. Building on Fricker’s work on epistemic injustice, scholars have identified and explained various kinds of epistemic injustice such as epistemic oppression (Dotson, 2014), silencing (McGlynn, 2019) and epistemic injustice in utterance interpretation (Peet, 2017). In the remainder of this subsection, I proceed to describe the epistemic vices of excess curiosity, gossip and excess inquisitiveness and illustrate how they relate to the concept of epistemic injustice.

Virtue responsibilists have categorized curiosity as an intellectual virtue (see e.g., Baehr 2011, pp. 19–21; Turri et al., 2017; Ross 2020). For instance, Ross (2020, p. 116) defines the intellectual virtue of curiosity as a character trait involving “the disposition to experience appropriately discerning, exacting and timely curiosity, motivated by a non-instrumental appreciation of epistemic goods”. I understand excessive curiosity as the corresponding excess-motivation vice of curiosity. Excess curiosity is an epistemic vice characterized by a lack of epistemic restraint (Manson, 2012). Consider, for example, the case of John. Being excessively curious, John does not hesitate to open the mail of his neighbors whenever the opportunity arises. To make matters worse, due to his unlimited ‘thirst’ for information, John is motivated to actively intercept the mails of his neighbors. He does not simply open the mail of other people whenever such letters find their way to him, but he actively seeks to intercept the letters of other people and read them before they reach their intended recipient. In other words, John is not able to restrain his epistemic desires even when they drive him to burden and/or cause harm to others. One’s personal correspondence could contain all sort of personal information. Mary might be expecting the results of her latest health check-up while Jack might be waiting for his tax records and bank statement. Having read their letters, John has obtained personal information about his neighbors without their consent. He has come to possess sensitive information which he was not warranted to obtain. Hence, he has caused epistemic harm to his neighbors—he has inflicted an injustice of an epistemic kind. He has undermined their right to privacy of information^6^.

Neither motivationalism nor obstructivism can account as to why excessive curiosity should be classified as an epistemic vice. The above example shows that excessive curiosity is not an epistemic vice due to a lack of epistemic desires or vicious epistemic motivations. John does not oppose or antagonize epistemic goods. Once he intercepts and reads the mail of his neighbors, he passes it on to them acting as if he has not read it. He does not deprive his neighbors of their mail—he simply reads it before they do. His actions stem from his strong epistemic desires^7^. Also, John’s excessive curiosity which leads him to read his neighbor’s mail does not obstruct the gaining, keeping, and/or sharing of epistemic goods. On the contrary, it seems to be conducive to the sharing of knowledge—if John were not overly curious, he would not actively seek to acquire information about his neighbors.

Gossip is another example of a non-obstructing, excess-motivation kind of intellectual vice (for more on the intellectual vice of gossip see Kotsonis 2021). Gossip involves the verbal transaction between two agents concerning a third agent with the sole purpose of acquiring ‘juicy’ information about the target’s personal life^8^. The disposition to gossip is an acquired character trait (i.e., one is a gossiper). Through gossip the agent comes to acquire epistemic goods which they are not warranted to possess. They come to violate the right of privacy of information of other people. Consider, for example, the case of Nick. Nick is an excellent gossiper—he is both epistemically motivated and competent in the activity of gossiping. Through gossiping with Andreas, who was in the past romantically involved with Nick’s boss Stephan, Nick acquires the truth about his boss’s love affairs (e.g., their choice of partners). In addition, through gossiping with Andreas, Nick also comes to learn the truth about Stephan’s financial situation (i.e., that he is rich) and mental health issues (i.e., that he suffers from panic attacks)^9^.

Gossip is neither attributable to imperfect epistemic motivations nor does it obstruct the acquisition, transmission, and retention of knowledge. The agent who possesses this trait is motivated to acquire epistemic goods through gossip and values those epistemic goods for their own sake (e.g., does not have some ulterior non-epistemic motivation for engaging in acts of gossip). In addition, through the activity characteristic of gossip, the excellent gossiper is in a position to acquire epistemic goods which would not be accessible to him through other means. Of course, one does not necessarily engage in acts of gossip out of their desire to acquire epistemic goods. For instance, one may engage in idle gossip to blend in with a group of people. However, for the purposes of this paper, I narrow my discussion to acts of gossip ultimately aiming at the acquisition of epistemic goods (i.e., acquiring ‘juicy’ information about another agent).

Besides gossip and excess curiosity, excess inquisitiveness is also another example of a non-obstructing, excess motivation epistemic vice. Watson (2015) has recently characterized inquisitiveness and accordingly classified it as a responsibilist kind of virtue. According to Watson (2015, p. 277), the agent who possesses the virtue of inquisitiveness is “characteristically motivated to engage sincerely in good questioning”. In other words, when one possesses the virtue of inquisitiveness, one has the competency to engage in good questioning whenever this is epistemically appropriate. I understand excessive inquisitiveness as the excess-motivation vice of the virtue of inquisitiveness. Due to their inability to exercise epistemic restraint, the agent who possesses excessive inquisitiveness has the disposition to engage in excessive questioning.

Consider the following example of excessive inquisitiveness: Gale, who is characterized by her disposition to engage in excessive questioning, has dinner with her best friend Harry and his new partner James. During dinner, and because of her tendency for excessive questioning that is characterized by a lack of epistemic restraint, Gale asks Harry several personal questions. Her questions are of such nature and phrased in such manner that they reveal things about Harry’s personal life—things that James was not aware of. These are things that Harry would not like to discuss with him at this point in their relationship. Imagine, for example, that she asks him whether he has any news regarding his latest health checkup and health problems. Or imagine that she asks him about his sister and the terrible relationship he has with her. Also, imagine that because Gale is his best friend and because he is kind-hearted, Harry responds to her questions—albeit in a rather reluctant and evasive manner. Still, Gale acquires epistemic goods through her excessive questioning—she learns about Harry’s health condition and the latest news about his relationship with his sister. Nonetheless, she has caused epistemic harm to her friend. Her inability to restrain her epistemic desire to acquire epistemic goods through persistent questioning has forced Harry to unwilling self-disclosure in the presence of his partner, James.

The vice of excessive inquisitiveness does not originate from imperfect epistemic motivations. On the contrary, Gale is strongly motivated to engage in questioning because of her strong epistemic desires. For example, she has a strong desire to know about Harry’s health condition. Also, excessive inquisitiveness does not obstruct the acquisition, transmission, and retention of knowledge. Through the characteristic activity of this vice, Gale is able to acquire certain epistemic goods. It could even be argued that Gale’s disposition for excessive questioning is also the reason that James has acquired certain truths about his partner Harry.

Perhaps one could argue that the structure of the above example is unique and that in most circumstances excessive questioning does not cause epistemic harm to other agents. However, I maintain that if one has the tendency for excessive questioning that is characterized by a lack of epistemic restraint and consideration for context, sooner or later they are going to cause epistemic (and potentially also non-epistemic) harm to other agents. Imagine, for example, a student who keeps asking her professor personal questions during lecture time. Also consider the case of Donald who decides to watch a movie with a friend who will not stop asking him questions about unrelated matters—Donald is bound to miss the opportunity to acquire certain information. Lastly, consider the case of Christin who, due to her excessive questioning, keeps distracting Helen from quietly reading her philosophy papers^10^.

### Non-obstructing, excessive-motivation epistemic vices: discussing objections

One could object to my argument that excessive curiosity, gossip, and excessive inquisitiveness should be classified as epistemic vice. As already noted, they do not entail imperfect epistemic motivations, nor do they obstruct the acquisition, retention, and transmission of epistemic goods. Hence, one could maintain that the theories of motivationalism and obstructivism cannot be used to identify them as epistemic vices simply because such traits are not vices of the intellect.

But assuming that they are not epistemic vices, how should then one classify traits such as excessive curiosity, gossip, and excessive inquisitiveness? Perhaps one could attempt to make the case that such qualities are characterized by moral instead of epistemic failings. One could argue that the trait of excessive curiosity can lead one to act in ways that are considered immoral. For instance, John is doing something morally wrong when he intercepts his neighbor’s correspondence. Also, the kind of gossip I am discussing in this paper involves the violation of norms by the speaker: they speak about topics that are considered taboo. Hence, one could argue that there is something morally wrong in talking about another person’s private life behind their back. Lastly, one could argue that excessive inquisitiveness involves acts which are morally wrong. For example, Gale, through her insistent questions, is verbally harassing Harry.

First, in reply to such an objection, one could highlight the fact that scholars who work in virtue responsibilism are reluctant to argue in favor of a sharp distinction between intellectual virtues/vices and moral ones. For example, Zagzebski (1996, p. 158) notes that we do not have any reason for categorizing intellectual virtues and moral virtues as distinct. Roberts and Wood (2007, p. 180) maintain that no strict line can be drawn between intellectual and moral virtues and Baehr (2011) argues that intellectual and moral virtues are not mutually exclusive^11^. In agreement, I argue that moral and intellectual virtues are not clearly distinguishable. They are quite similar both in their nature and the manner in which agents come to acquire them. Both intellectual and moral virtues involve handling of feelings, proper understanding of the world and a degree of voluntariness (for more on this point, see Zagzebski 1996). In addition, they are both acquired through imitation and habituation. There are even some virtues, such as courage and humility, that have “both moral and intellectual forms” (Zagzebski, 1996, p. 159). Therefore, as Watson (2015, p. 280) rightly argues, since moral and intellectual virtues cannot clearly be distinguished then it is only to be expected that cases of intellectual vices can be cashed out in terms of moral vices as well. In other words, the fact that the examples of excess-vices discussed in this paper can also be understood in terms of moral vices does not constitute an objection to my arguments.

However, this might not provide a satisfactory answer to some who would push on with the idea that the kind of epistemic vice that I discuss in this paper is better understood in terms of non-epistemic consequences. To answer this, following Cassam’s (2019) consequentialist understanding of epistemic vice, I maintain that such traits primarily qualify as epistemic vices because they are fundamentally characterized by the negative epistemic consequences which they systematically bring about (although they are not of the kind of epistemic consequences that Cassam has in mind when proposing the theory of obstructivism). Therefore, in a sense, they fall under the theory of obstructivism when such theory is broadly understood as conceptualizing epistemic vices in terms of their negative epistemic consequences.

As already pointed out, there is an injustice of an epistemic kind systematically inflicted through the characteristic activities of these traits. All three epistemic vices discussed in this paper stem from a lack of epistemic restraint—and this lack of epistemic restraint is the reason that the agents who possess these traits cannot control their epistemic desires and end up causing epistemic harm to other agents (e.g., acquiring and/or revealing their personal information without their consent^12^^,^ 13). The harm caused is epistemic, as opposed to non-epistemic, because the harm is *knower-initiated* (i.e., caused by the knower to another agent the moment the knower comes to acquire epistemic goods), *involves epistemic goods* (someone’s private information) and the agent who suffers the epistemic injustice is *harmed in their capacity as a knower* (they are deprived of the choice to be the sole knower of their personal information and disclose them only to whomever and whenever they desire/deem fit)^14^. This fits well with Pohlhaus’ viewpoint on epistemic injustice according to which epistemic injustice not only “wrongs a knower as a knower, but also is a wrong that a knower perpetrates as a knower” (Pohlhaus, 2017, p. 14). Note also that the epistemic harm caused by the characteristic activities of these traits does not hinge on the infliction of non-epistemic harm. For instance, in the example of excessive curiosity discussed previously, John causes epistemic harm (when he opens his neighbor’s letters) independently of whether he use the information he acquires to cause further harm (as for example if he uses it for extortion). He causes epistemic harm to them *the moment* he reads their personal correspondence and obtains information about their personal life without their consent. If John decides to let everyone know that Jack has a lot of money, he may also cause non-epistemic harm to him. People, for example, might start asking Jack for a loan and begrudging him when he does not give them one. However, even though John may decide not to do anything with the information he obtains, his excessive curiosity has caused epistemic harm.

Some may have objections to my characterization of excessive curiosity, gossip, and excessive inquisitiveness as epistemic vices, even when they bear the fruit of knowledge. Consider, for instance, the following case: A scientist has an overweening urge to learn the truth. This leads them to invade the privacy of their colleagues, reading their results. As a result of their obsessive inquiry, the scientist makes an epochal breakthrough in her understanding of the universe. Acknowledging the great epistemic benefits that flowed from their snooping, does the scientist really display epistemic vice in reading their colleagues’ results, simply because she has invaded their privacy? Indeed, one could argue that the epistemic benefits of the scientist’s snooping outweigh the harms. Still, I maintain that this does not undo the fact that they are displaying an epistemic vice: they have caused epistemic harm to their colleagues by invading their privacy without their consent. The kind of traits that I am discussing in this paper are fundamentally characterized by the epistemic harm which they *systematically* bring about, and the fact that there are some *extreme* cases in which the positive results might outweigh the harms does not undo my overall argument. Similarly, one could come up with cases in which (due to some stroke of luck) vices such as gullibility, dogmatism, epistemic cowardliness, and carelessness bring about positive epistemic outcomes. Still, following obstructivism, this does not mean that we should not categorize such traits as epistemic vices. It remains that in the *vast majority of cases* these traits will obstruct the gaining, keeping or sharing of knowledge.

Perhaps one could take one step back and instead argue (against my view) that the traits discussed in this paper are not vices of any kind. In the previous paragraph, I highlighted how the traits discussed in this paper can be classified under Cassam’s (2019) broad consequentialist understanding of epistemic vice—the agent who possesses such traits systematically causes epistemic harm to other agents through the characteristic activities of these traits. This also relates to Foot’s (1978) Neo-Aristotelian conception of virtue and vice. According to Foot (1978), virtues are beneficial characteristics that an agent needs to possess for both her sake and those around her. Correspondingly, following Foot’s conceptualization of virtue, one could conceive of vices as those traits that systematically cause harm to the agent and/or others. Hence, excessive curiosity, gossip and excessive inquisitiveness should be classified as (epistemic) vices since they clearly cause (epistemic) harm to those around the agent.

## Altering the theories of obstructivism and motivationalism

In this section, I propose ways in which the theories of motivationalism and obstructivism can be altered in such a manner as to account for non-obstructing, excess-motivation vices. I begin by discussing obstructivism and argue that the theory’s understanding of harmful epistemic consequences can be broadened to include non-obstructing negative epistemic consequences. I then proceed to discuss motivationalism and propose a way in which the theory can be expanded to include imperfect epistemic motivations of an excessive kind.

### Obstructivism and non-obstructing epistemic vices

As already noted, Cassam (2019) puts forward a consequentialist view on vice. For him, vices “systematically obstruct the gaining, keeping or sharing of knowledge” (Cassam, 2019, p. 1). However, none of the epistemic vices discussed in the previous section bring about such kind of negative epistemic consequences. Excessive curiosity, gossip and excessive inquisitiveness do not systematically obstruct the gaining, keeping, and/or sharing of knowledge. On the contrary, they are all conducive to effective epistemic inquiry—i.e., the agent who possesses these traits is in a better epistemic position to acquire epistemic goods than the agent who lacks such qualities^15^. Hence, as already discussed, obstructivism (in its current form) does not account for the kind of epistemic vice discussed in the previous section.

Still, in this short subsection, I want to point out that with some theoretical alterations the theory of obstructivism can easily incorporate non-obstructing epistemic vices under its understanding of the concept of vice. As it has been already noted, in its broader version, obstructivism upholds that all intellectual vices are to be understood in terms of the negative epistemic consequences they bring about. Expanding on Cassam’s (2019) obstructivism theory, one could argue that epistemic vices are character traits, epistemic attitudes, and ways of thinking that systematically obstruct the acquisition, transmission, and retention of knowledge *and/or cause other kinds of epistemic harm*. A more general phrasing of this version would be the following: *epistemic vices are character traits, attitudes, or ways of thinking that systematically cause epistemic harm to the agent who possesses them and/or other agents.*

This version of obstructivism retains all (or at least most) essential characteristics of Cassam’s (2019) view on vice and accounts for those epistemic vices which the original version is not broad enough to include. Let us briefly consider how, when broadened in this manner, the theory of obstructivism accommodates for non-obstructing, excess-motivation epistemic vices. Excessive curiosity, gossip and excessive inquisitiveness are blameworthy, or otherwise reprehensible qualities because of their negative epistemic consequences. Although they do not obstruct the acquisition and retention of knowledge, they nonetheless cause other kinds of epistemic harm. The agents who possess such traits cause epistemic harm to other people through the characteristic activities of these vices.

One might point out that the way I have defined epistemic vice does not justify me in presenting this altered view as an obstructivist one. It is true that calling obstructivist a viewpoint which includes non-obstructing vices is counterintuitive. Perhaps it would be better to go back to a more generic description and describe this view on vice as epistemic consequentialism. This would reflect that epistemic vices are characterized by their negative epistemic consequences (which are not limited to knowledge-obstruction). Still, irrespectively of how one calls this new version, the most fundamental aspects of Cassam’s (2019) obstructivist view remain. For example, similarly to the original version of the theory, this new version upholds that epistemic vices (1) are not limited to traits but include various attitudes and ways of thinking, (2) are characterized by their negative epistemic consequences, (3) do not require vicious and/or deficient motivational states and (4) reflect badly on the agent and the agent can be criticized for possessing them (Cassam, 2019, pp. 22–23).

### Motivationalism and excess-motivation vices

Unlike obstructivism, perhaps it can be insisted that (in its current form) motivationalism can account for excess-motivation virtues. As already noted, motivationalism is the view according to which all kinds of epistemic vices are attributable to motivations which lack epistemic value either because they are deficient or because they are outright vicious. Hence, one could put forward the idea that excessive curiosity, gossip and/or excessive inquisitiveness are characterized by a lack of epistemic motivation, a motivation which does not perceive epistemic goods as goods in themselves and/or even an explicit dislike for epistemic goods. Nonetheless, I do not believe that this is the case for either of these three vices. As shown in Sect. 3, the agents who possess the vices of excessive curiosity, gossip and/or excessive inquisitiveness are motivated to act out of their desire for epistemic goods. None of these virtues require that the agent is motivated by imperfect epistemic desires (at least not in the way motivationalism currently understands imperfect motivational states, i.e., as involving either deficient epistemic drives or vicious ones). Hence, it seems safe to conclude that (in its current form) motivationalism does not account for the kind of epistemic vices discussed in this paper^16^.

Nonetheless, I want to highlight the fact that motivationalism has a way to explain vices such as excessive curiosity, gossip and excess inquisitiveness in terms of imperfect epistemic motivations—the agent who possesses these vices lacks epistemic restraint and hence their epistemic desires become unregulated and excessive (in the sense that they cause epistemic harm)^17^. However, to support such view, the theory of motivationalism will need to be altered in such a manner as to include excessive epistemic desires as cases of imperfect epistemic motivations. The way that motivationalism is currently developed in the literature of vice epistemology does not allow room for cases of excessive epistemic desires. For example, Montmarquet (2000, pp. 138–139) understands intellectual vices as involving a lack of epistemic effort, Baehr (2010) puts forward the idea that epistemic vices are characterized by the agent’s lack of epistemic drives and Tanesini (2018, p. 350) maintains that “intellectual character vices involve non instrumental motives to oppose, antagonise, or avoid things that are epistemically good in themselves”.

In order to characterize the vices that I have discussed in this paper in terms of motivation, one would first have to accept that there is such a thing as epistemic desires of an excessive kind. One might be inclined to argue (against my view) that epistemic desires can never be of an excessive nature- and uphold that the stronger the epistemic desires one has, the better^18^. The following example is a good way to illustrate the existence of excessive epistemic desires and the negative epistemic (and non-epistemic) consequences stemming from such desires. Nicol is an exceptionally capable person when it comes to epistemic pursuits. She is smart and driven in her epistemic endeavors. Still, Nicol has also a strong desire to acquire the truth about various conspiracy theories—her desire is so strong that it could even be understood as an obsession^19^. Because of this obsession (and the time and effort she puts into trying to find the truth), she drops out of college, ends up having no friends, rarely sees her family and lives below the poverty line. Had Nicol being less interested in acquiring the truth, she would be living a much better life (which would also perhaps allow her to acquire more epistemic goods than she does now—e.g., by going to college). On top of that, because of her excessive epistemic desires, Nicol causes epistemic harm to other agents. For example, believing that her neighbors are spies, she comes to intercept and read their personal correspondence.

Assuming that my overall argument in this paper stands (i.e., that current versions of motivationalism cannot account for the epistemic vices of excess curiosity, gossip and excess inquisitiveness), scholars working in motivationalism have two main options: (i) bite the bullet, (ii) alter motivationalism so as to incorporate such epistemic vices. In this short subsection, I have tried to flesh out what the latter response might look like^20^.

## Concluding remarks

My aim in this paper has been to present and discuss a novel understanding of the nature of epistemic vice. I argued that there are certain epistemic vices (i.e., excess curiosity, gossip and excess inquisitiveness) of a non-obstructing excess-motivation kind, that neither the theory of motivationalism nor the theory of obstructivism are broad enough to include under their conceptualization of the term of intellectual vice. Throughout this paper, I illustrated how these vices relate to the concept of epistemic harm and characterized them both in terms of negative epistemic consequences and excessive epistemic motivations. To remedy for the inability of motivationalism and obstructivism to account for such vices, I developed and proposed broader versions that retain the essential characteristics of these theories and include non-obstructing, excess-motivation epistemic vices under their understanding of the concept of intellectual vice.

## Footnotes

1 Throughout this paper, I use the terms ‘intellectual virtue’ and ‘epistemic virtue’ as well as the terms ‘intellectual vice’ and ‘epistemic vice’ interchangeably.

2 I should note that although I identify such epistemic vices as traits, I follow Cassam’s (2019) conceptualization of epistemic vice in that I believe that excess curiosity, gossiping and excess inquisitiveness can also be understood in terms of epistemic attitudes and/or modes of thinking. For instance, one’s attitude and/or way of thinking could be characterized as a gossiping one—i.e., characterized by a desire to acquire ‘juicy’ information (e.g., choice of sex partners) about another person behind their back.

3 This relates to the concept of violence (which can be understood as including non-physical harm) and the view that violence is inevitably intertwined with morality—see Magnani 2011.

4 Later in this section, I will be formulating arguments that rely on a reverse version of Fricker’s (2007) conception of epistemic injustice. According to this reverse version, there are cases of epistemic injustice in which the knower inflicts epistemic harm to other people.

5 Fricker’s (2007) analysis of epistemic injustice is not without its critics. For instance, Maitra (2010, p. 210) has argued that the two kinds of epistemic injustice discussed by Fricker “are more similar than she allows”.

6 For more on a person’s right to privacy of information, see the principles of information privacy as well as privacy laws (e.g., EU’s General Data Protection Regulation and the Data Protection Directive). It is the kind of information protected by such laws that I am referring to here.

7 Ross (2020, p. 116) argues that “an agent’s curiosity will be virtuous…to the extent that it is non-instrumentally motivated by their appreciation of epistemic goods”. John’s curiosity satisfies this condition: he is motivated to act out of his love for epistemic goods. His curiosity does not involve deficient or vicious epistemic motivations.

8 According to Robinson (2016), there are two different kinds of gossip: (i) evaluative and (ii)idle. The former involves normative evaluations of the target of gossip (e.g., George is a cheater, Mary is dishonest), while the latter does not. In addition, both kinds of gossip are characterized by a violation of norms. In the case of evaluative gossip, the subject of gossip has violated a certain set of norms (e.g., moral norms, cultural norms) while in the case of idle gossip it is the gossiper that violates norms by talking about topics which are considered taboo (e.g., talking about his boss’s sex life). For more on the topic of gossip, see also Lind et al., (2007), Bertolotti & Magnani (2014), and Alfano & Robinson (2017). Note that Robinson (2016) and Alfano & Robinson (2017) focus on evaluative gossip, while in this paper I am focusing on idle gossip which ultimately aims at the acquisition of ‘juicy’ information about the personal life of the subject.

9 One may point out that gossip is often unreliable. However, the excellent gossiper is, in the vast majority of cases, in a position to judge when one of their interlocutors is lying to them. After all, being themselves an excellent gossiper, they are aware of the fact that information acquired via gossip can often be unreliable and hence are always careful to double-check them (e.g., by gossiping about the same piece of information with other agents).

10 Perhaps one could argue that some of these cases can be understood in obstructivist terms. For instance, in the film example, one’s excessive questioning can obstruct another agent’s acquisition of knowledge. Still, the problem is that obstructivism accounts only for some of these cases. As already noted, for example, in Gale’s case there is on obstruction of epistemic inquiry. Hence, as I will argue in section IV, the scope of obstructivism needs to be broadened in order to account for all kinds of epistemic harm inflicted by vices such as excessive inquisitiveness (and not simply epistemic harms of an obstructing nature).

11 Note that my discussion at this point has been greatly informed by Watson’s (2015) discussion of how contemporary virtue scholars understand the relation between moral and intellectual virtues.

12 All three traits trample on the notion of privacy of information. Still, it should be noted that the agents who possess these traits are not motivated to act out of a desire to cause epistemic harm (violate a person’s right) but out of their desire to acquire epistemic goods—i.e., their actions originate from good epistemic motivations.

13 Some might point out that one’s right to privacy of information should be understood as an ethical right. Still, this does not necessarily preclude us from understanding the violation of this right as bringing about epistemic harm. I have argued that the harm caused is epistemic, as opposed to non-epistemic, because the harm is *knower-initiated* (i.e., caused by the knower to another agent the moment the knower comes to acquire epistemic goods), *involves epistemic goods* (one’s private information) and *the agent is wronged qua knower* (they are deprived of the choice to be the sole knower of their personal information and disclose them whenever they themselves desire/deem fit).

14 One might argue that these conditions are not sufficient since they seem to suggest that blackmail is an epistemic harm: it is knower-initiated and involves epistemic goods. Still, I maintain that blackmail does not fit the description. Blackmail requires coming to acquire another agent’s private information (and sometimes not even that—one could bluff) without (or even with) their consent and *then explicitly using this information to gain some benefit* (which may or may not be of epistemic nature and in most cases isn’t). In the cases I discuss in this paper, agents do not need to use the information they obtain (in any way) for epistemic harm to take place—epistemic harm occurs the moment they acquire another agent’s information without their consent and the harm caused does not hinge on any additional actions and/or consequences.

15 The discussion can be expanded to include thinking vices (Cassam, 2019); and traditional “fallacies” that have positive cognitive value (Woods, 2013; Magnani, 2015).

16 To be fair, Battaly (2010) has discussed cases of excessive epistemic desires and has linked them to epistemic vice. Still, it seems that she is one of the very few scholars to have made this connection. Most scholars following the motivationalism viewpoint are either reluctant to accept that there is such a thing as excessive epistemic desires or have not considered it as a possibility (for more information, see the next paragraph and footnote 13).

17 To give an example, the vice of excessive curiosity can be understood as involving unregulated and excessive epistemic drives, while the virtue of curiosity can be understood as involving restraint, regulation, and control of epistemic desires on the part of the agent.

18 For instance, Zagzebski (1996, pp. 196–197) seems somewhat reluctant to attribute vices such as the ones discussed in this paper to an excessive motivation to know since she believes that such motivation is not a defect and does not necessarily give rise to vice. Also, Code (1987, pp. 44–45) may be hesitant to attribute “intellectual virtue to the voracious collector of facts” but still, her hesitancy does not stem from the belief that epistemic desires can be of an excessive nature but from the view that “it is possible, theoretically at least, to know everything, yet understand nothing”.

19 One could argue that what is wrong with agents such as Nicol is not that they care about epistemic goods too much but that they care about the wrong things. However, this does not sound right. Being motivated to find out the truth about things such as the JFK assassination, 9/11 and the Covid-19 pandemic is not caring about the wrong things—some of these events have had a tremendous impact on our lives.

20 It might also be worthwhile to note that the idea that there are both excessive and deficient levels of epistemic motivations fits well with the Aristotelian conception of the golden mean (see, e.g. *EN*, 1107a). For Aristotle, virtue lies in a mean between two vices and since virtues and vices are partly constituted of motivations, the idea that motivational states admit to both excess and deficiency seems to follow. This might be of some sway for scholars (working in motivationalism) who maintain that they build their conceptions of intellectual vice based on Aristotle’s conceptualization of the term (see e.g., Zagzebski 1996; Battaly, 2014, 2017).

## References

[CR1] Alfano M, Robinson B (2017). Gossip as a burdened virtue. Ethical Theory and Moral Practice.

[CR2] Baehr J (2009). Evidentialism, vice, and virtue. Philosophy and Phenomenological Research.

[CR3] Baehr J, Battaly H (2010). Epistemic malevolence. Virtue and vice: Moral and epistemic.

[CR4] Baehr J (2011). The inquiring mind.

[CR5] Battaly H (2010). Epistemic self-indulgence. Metaphilosophy.

[CR6] Battaly H, Matheson J, Vitz R (2014). Varieties of epistemic vice. The ethics of belief.

[CR8] Battaly H, Kidd I, Medina J, Pohlhaus G (2017). Testimonial injustice, epistemic vice, and vice epistemology. The Routledge handbook of epistemic injustice.

[CR9] Bertolotti T, Magnani L (2014). An epistemological analysis of gossip and gossip-based knowledge. Synthese.

[CR10] Cassam Q (2016). Vice epistemology. The Monist.

[CR11] Cassam Q (2019). Vices of the mind: From the intellectual to the political.

[CR12] Code L (1984). Toward a responsibilist epistemology. Philosophy and Phenomenological Research.

[CR13] Crerar C (2018). Motivational approaches to intellectual vice. Australasian Journal of Philosophy.

[CR14] Dotson K (2014). Conceptualizing epistemic oppression. Social Epistemology.

[CR15] Driver J (2001). Uneasy virtue.

[CR16] Foot P (1978). Virtues and vices and other essays in moral philosophy.

[CR17] Fricker M (2007). Epistemic injustice: Power and the ethics of knowing.

[CR36] Kotsonis, A. (2021). The curious case of the excellent gossiper. *Philosophia*. 10.1007/s11406-021-00444-1

[CR18] Lind PG, Silva D, Andrade LR, Herrmann HJ (2007). Spreading gossip in social networks. Physical Review E.

[CR19] Magnani L (2011). Understanding violence: The intertwining of morality, religion, and violence: A philosophical stance.

[CR20] Magnani L (2015). Naturalizing logic: Errors of reasoning vindicated: Logic reapproaches cognitive science. Journal of Applied Logic.

[CR21] Maitra I (2010). The nature of epistemic injustice. Philosophical Books.

[CR22] Manson N (2012). Epistemic restraint and the vice of curiosity. Philosophy.

[CR23] McGlynn A (2019). Testimonial injustice, pornography, and silencing. Analytic Philosophy.

[CR24] Montmarquet J (1993). Epistemic virtue and doxastic responsibility.

[CR25] Montmarquet J, Axtell G (2000). An ‘internalist’ conception of virtue. Knowledge, belief, and character: Readings in virtue epistemology.

[CR26] Peet A (2017). Epistemic injustice in utterance interpretation. Synthese.

[CR27] Pohlhaus G, Kidd I, Medina J, Pohlhaus G (2017). Varieties of epistemic injustice. The Routledge handbook of epistemic injustice.

[CR28] Robert R, Woods WJ (2007). Intellectual virtues: An essay in regulative epistemology.

[CR29] Robinson B (2016). Character, caricature, and gossip. The Monist.

[CR30] Ross L (2020). The virtue of curiosity. Episteme.

[CR31] Tanesini A (2018). Epistemic vice and motivation. Metaphilosophy.

[CR32] Turri, J., Alfano, M., & Greco, J. (2017). Virtue epistemology. In E. N. Zalta (Ed.), *Stanford encyclopedia of philosophy*, https://plato.stanford.edu/entries/epistemology-virtue/

[CR33] Watson L (2015). What is inquisitiveness. American Philosophical Quarterly.

[CR34] Woods J (2013). Errors of reasoning. Naturalizing the logic of inference.

[CR35] Zagzebski L (1996). Virtues of the mind: An inquiry into the nature of virtue and the ethical foundations of knowledge.

